# Predicting floods with Flickr tags

**DOI:** 10.1371/journal.pone.0172870

**Published:** 2017-02-24

**Authors:** Nataliya Tkachenko, Stephen Jarvis, Rob Procter

**Affiliations:** 1 Warwick Institute for the Science of Cities, University of Warwick, Coventry, CV4 7AL, United Kingdom; 2 Department of Computer Science, University of Warwick, Coventry, CV4 7AL, United Kingdom; Bristol University/Remote Sensing Solutions Inc., UNITED STATES

## Abstract

Increasingly, user generated content (UGC) in social media postings and their associated metadata such as time and location stamps are being used to provide useful operational information during natural hazard events such as hurricanes, storms and floods. The main advantage of these new sources of data are twofold. First, in a purely additive sense, they can provide much denser geographical coverage of the hazard as compared to traditional sensor networks. Second, they provide what physical sensors are not able to do: By documenting personal observations and experiences, they directly record the impact of a hazard on the human environment. For this reason interpretation of the content (e.g., hashtags, images, text, emojis, etc) and metadata (e.g., keywords, tags, geolocation) have been a focus of much research into social media analytics. However, as choices of semantic tags in the current methods are usually reduced to the exact name or type of the event (e.g., hashtags ‘#Sandy’ or ‘#flooding’), the main limitation of such approaches remains their mere nowcasting capacity. In this study we make use of polysemous tags of images posted during several recent flood events and demonstrate how such volunteered geographic data can be used to provide early warning of an event before its outbreak.

## Introduction

Contemporary environmental hazard warning systems, based on highly evolved and specialised forecasting procedures, have replaced people’s reliance on their personal observations and intuition. Nowadays, several types of data can be collected to assist specialists to predict where and when an environmental hazard might occur. For instance, monitoring the amount of rainfall occurring on a realtime basis or the rate of change in river stage can help indicate the severity and immediacy of the threat of flooding [1]. The recent proliferation of digital social media platforms have introduced a new and additional source of information to be taken into account when designing warning systems and planning their implementation [2]. Thus, the US Geological Survey (USGS) was the first environmental institution to recognise the value of such user generated content (UGC), recognising that analysis of the content and geographic distribution of Twitter postings—i.e., ‘social sensors’– can be a useful supplement to instrument-based estimates from physical sensors of earthquake location and magnitude [3]. A more recent study reported on the value of the Flickr image sharing platform in nowcasting of the evolving ex-hurricane Sandy, where aggregated volumes of UGC was found to replicate air depression fluctuations over the same time period [4].

Various open user generated content is therefore now widely recognised as a valuable nowcasting tool in the conditions where official observation stations, sensors or gauges do not provide sufficient geographic coverage to be able to generate timely and spatially accurate updates about situation on the ground. In addition to precise spatial information, content and various metadata elements can provide information on people’s reactions and behaviours, the magnitude of the event, etc., which may serve as timely intelligence for directing the efforts of rescue services.

Despite the growing interest, current methods of using social media data have begun to reach the limits of their potential; this can be explained primarily by the ways in which ‘useful’ data components are being defined. For example, all current analyses are based on the exact words and word-combinations designating either type of a hazard itself (e.g., ‘flood’, ‘hurricane’) or its name (e.g., ‘Sandy’, ‘Katrina’) [5, 6]. Whilst useful for operational purposes, from the forecasting perspective they hold little value and so current frameworks are limited to their nowcasting capacity.

Following a language-based approach, we turned our attention to the domain of environmental semantics, which defines the verbal structures people use in order to express their interactions with the natural environment [7]. We hypothesise that any natural phenomenon can be described by a much wider spectrum of words and structures than those that are currently being employed by analysts. Thus environmental anthropologists recognise that people’s engagement with, for example, water in the landscape, can manifest itself as a material artefact that features in their lives in a myriad of ways, many of which involve close sensory interaction [8, 9]. The natural landscape is seen as a cognitive experience [10, 11] of endlessly changing states, where, for example, water moves between oppositional extremes of a roaring flood and a still pool. Each of these states has its own qualities and is imbued with its own meanings, and all are always there in potential. Human sensory experience of these qualities is to some degree universal and this commonality contributes to the recurrent themes of meaning encoded in the natural landscape in many different contexts [12].

In this study we therefore aim to test the novel hypothesis that alternative environmental semantics can be linked to extreme environmental events and may serve as a predictor of an evolving hazard. We use flooding as a test case scenario.

## Materials and methods

### Data

We used the Yahoo Flickr Creative Commons 100M (YFCC100M) dataset containing a list of photos and videos uploaded to the Yahoo! Flickr platform between April 2004 and August 2014. All the audio-visual material provided in this database is licensed under one of the Creative Commons copyright licenses (CC:BY) [13].

Yahoo! Flickr has been previously identified as a social media platform capable of capturing an event outbreak with high precision. For example, recent research insights into the same dataset suggest that the uploading of topical content to Flickr can serve as an indicator of the outbreak of political unrest in any part of the world, irrespective of the language used [14]; a similar study has established a relationship between the air pressure trendline and postings to Yahoo! Flickr throughout ex-hurricane Sandy [4]. In contrast to Twitter or Facebook, which serve predominantly as tactical and strategic operations platforms to manage events and their posterior consequences [15–18], Yahoo! Flickr seems to exhibit significant potential as a nowcasting tool in conditions of absent or insufficient sensor or media coverage.

**Fig 1:**
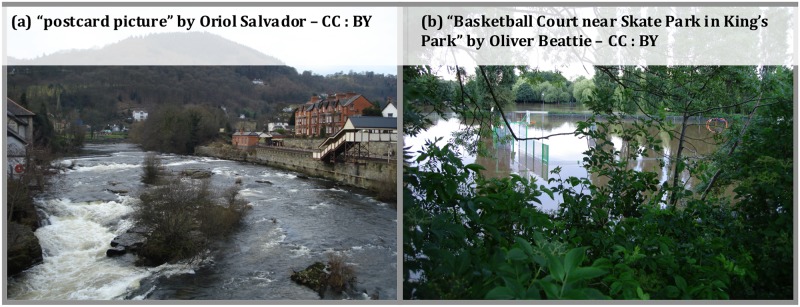
Two cases of the tag ‘river’ used with Flickr images: (a) capturing the scenic nature of a rocky, riverine environment and (b) illustrating water levels during a flood event.

This study aims to expand nowcasting social media analytics approaches towards the possibility to detect an event before its outbreak. We build our hypothesis on the fact that UGC, apart from the actual event-denoting tags, can also contain semantically approximated elements, capable of providing insight into an event before it reaches its peak. Thus, the tag ‘river’ can be equally seen in the context of scenic water landscape and flooded areas alike: Whilst holding the same neutral sentiment from the data mining perspective, its connotative qualities can be very different—depending on the context (Fig 1).

**Fig 2:**
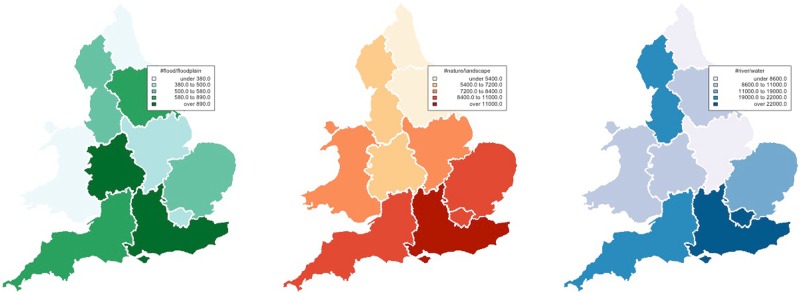
Spatial distribution of images with environmental and hydrological thematics uploaded to Flickr over the period 2004–2014 in England.

Flooding is a natural phenomenon that concerns the interface between land and water [5]; we selected from the Yahoo! Flickr dataset photographic and video material containing tags designating riverine environments: ‘landscape’, ‘nature’, ‘river’ and ‘water’. Their spatial distribution in the regions of England is presented in Fig 2.

**Fig 3:**
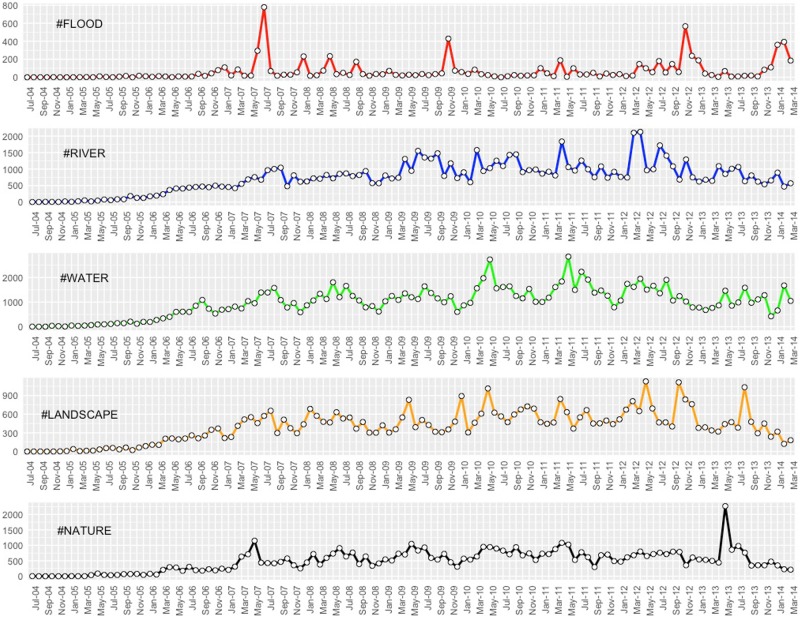
Monthly aggregated temporal profiles of the main environmental tags on Flickr (2004–2014).

The main characteristic of this dataset that is of interest for our study—the temporal behaviour of the selected tag elements (Fig 3)—was subsequently used to evaluate empirically the dependencies between neutral (‘river’, ‘water’, ‘nature’, ‘landscape’) and risk-signalling (‘flood’, ‘flooding’, ‘floodplain’) semantic elements.

### Methods

**Fig 4:**
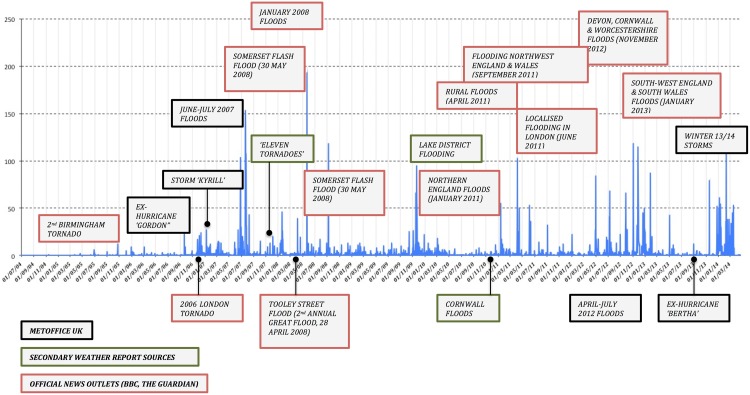
Timeline of Flickr activity during a number of flood events (2004–2014) of various magnitude, captured by official, secondary and mass media sources.

We began our method development with the novel hypothesis that polysemous tags and keywords exhibit forecasting behaviour in the context of an evolving weather event. During a certain time period before its peak, we argue that their semantics becomes distinct from the general context meaning [7] and acquires new properties under the influence of certain environmental factors, such as, for instance, observations of saturated soil or raised water levels in nearby streams. Because the primary aim of our study was to test the forecasting potential of social media platforms on their own (e.g., without complementary use of the environmental data), we therefore algorithmically defined the ‘buffer’ time period around each identified flood peak (Fig 4) (e.g., period before and after the peak of the event as reflected by the daily volume of pictures uploaded to the Yahoo! Flickr platform) as five days; however, there is scope for future data inquiries to adapt such periods for variable socio-environmental conditions:

$$\begin{array}{c} B_{peaks}={\left(\frac{N_{all}-N_{peaks}}{N_{peaks}}\right)}^{\frac{1}{2}} \end{array}$$ (1)

To identify whether the Yahoo! Flickr dataset contains elements with forecasting capacity vis-a-vis developing flooding events, we use a Deconstructed Cascade Correlation Matrix (DCCM), based on the algorithm proposed by Fahlman and Lebiere in the early 90s [17]. The main advantage of this supervised learning method lies in the fact that it overcomes the moving target problem by adding new hidden units and freezing their input-side weights, thus preventing a constantly changing environment. Due to its manoeuvrability and ease of deployment, DCCM has found wide application in multidisciplinary contexts, ranging from weather prediction to civil engineering applications. To adapt it to new applications, several new methods have been recently developed, notably Evolving Cascade Neural Networks (ECNN, 2003) [18] and Modified Cascade Correlation Neural Networks (MCCNN, 2010) [19].

The choice of DCCM in our study was motivated by three main factors: (a) Its flexibility, or vertical de-construction capacity (it allows algorithm decomposition into a number of intermediate subsystems, which, in our case, may potentially hold additional value for subsequent platform data analytics); (b) Its capacity for horizontal de-construction, i.e., to perform as an advanced regression model, whilst retaining the performance metric of each design variable; and finally (c) Its capacity to perform on-line learning, which adds value for subsequent algorithm integration into real-world forecasting systems.

We started our network with four selected input tags (‘nature’, ‘landscape’, ‘river’, ‘water’), plus two additional aggregated tags (‘RW’ and ‘NL’). Each of these inputs was connected to a single output (combined risk-signalling tags ‘flood’, ‘flooding’, ‘floodplain’), without any initial weight attribution. As an activation function we used a simple Pearson Product-Moment Correlation, which provides an advantage for our case study where linear output units are required:

$$\begin{array}{c} \rho_{X,Y}=\left(\frac{cov(X,Y)}{\sigma_{X}\sigma_{Y}}\right) \end{array}$$ (2)

**Fig 5:**
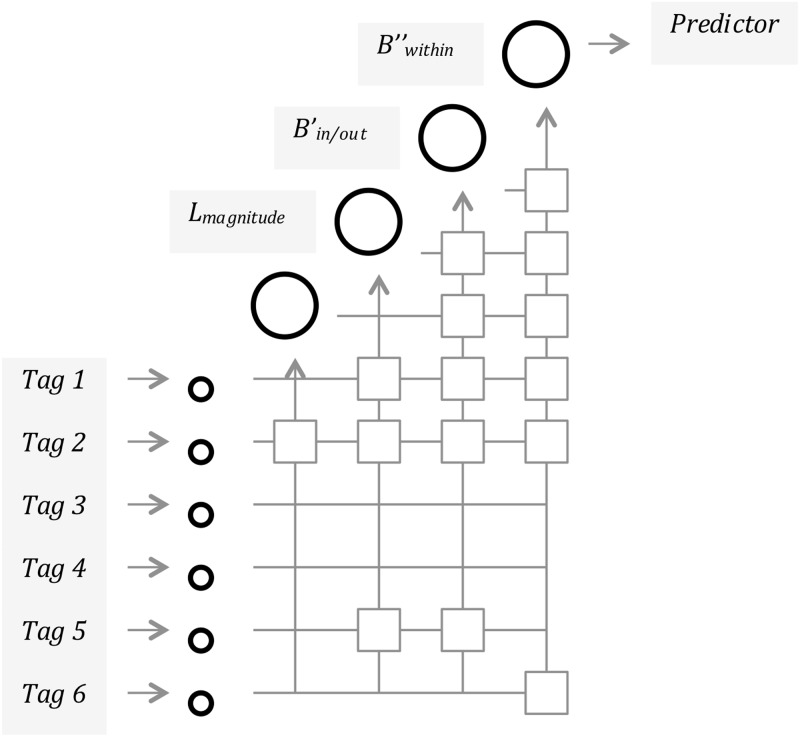
Cascade correlation Matrix workflow, adapted to identify Yahoo! Flickr tags with the best flood forecasting capacity.

Three conditional units are represented by the same output variable, each pre-processed (i.e., temporally deconstructed) to capture three main phenomena of interest: (a) Event magnitude; (b) Behavioural profiles within and outside flood peak periods (local maximal values of the video and photographic upload to the Yahoo! Flickr platform plus-minus five days before and after the spike); and (c) Actual behavioural trends within the flood peak periods. Once the routine is triggered by the first conditional unit, each new activated hidden unit receives a connection from each of the network’s original inputs (i.e., simple and aggregated tags) and also from every pre-existing hidden unit. Each conditional unit therefore adds a new layer to the network, which pushes its best performing predictors, until the best one(s) are identified (Fig 5).

Whilst design of this framework is primarily focused on the performance of each individual design variable (i.e., ‘tag’), it also accounts for their interaction when selecting hidden units to take forward before choosing the best possible predictor (a.k.a. ‘hidden candidate unit’). Therefore, whilst configurable and expandable, this machine learning algorithm primarily aims here to visualise which elements hold semantic-temporal relations with risk-signalling tags (e.g., ‘flood’ and ‘flooding’) and how those relations change as the events evolve.

## Results

**Fig 6:**
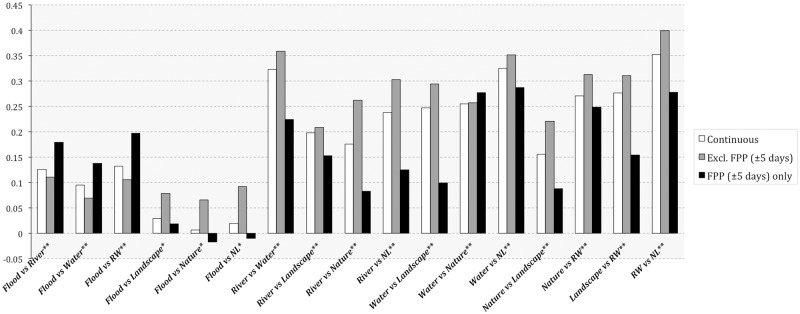
PPMCC values of the cross-dependencies between generic environmental, hydrological and risk-signalling semantic tags on the Yahoo! Flickr platform (2004–2014).

Fig 6 illustrates the dependency behaviours of tags inside and outside flood peak periods, without taking into account the magnitude of the event. These results clearly demonstrate that flood-related tags tend to correlate with hydrologically themed tags, which occur in the metadata of the uploaded content in isolation (e.g., ‘river’, ‘water’) or in combination (‘RW’). These dependencies are generally in contrast with how other tags, designating more generic environmental thematics, such as ‘nature’, ‘landscape’, relate to the risk-signalling semantics. Interestingly, ‘river’ and ‘water’ tags occupy somehow an intermediate position between the topic of natural hazard and more generic natural landscape theme: thus, we can observe strong correlations between ‘river’, ‘water’, ‘RW’ and ‘nature’, ‘landscape’, ‘NL’ tags in general, which, however, differ significantly within and outside flood peak periods. This decrease is simultaneously accompanied with strengthened relations between ‘flood’ and ‘water’, ‘river’ and ‘RW’ tags over the same time periods.

**Fig 7:**
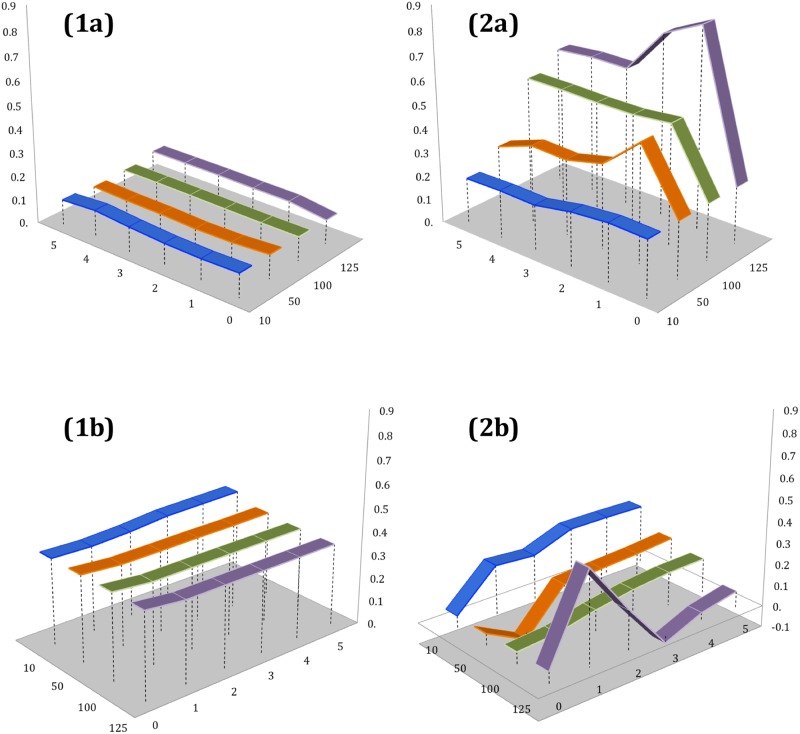
PPMCC values of the cross-dependencies between aggregated tag material (a) ‘RW’ vs. ‘flood’ and (b) ‘RW’ vs. ‘NL’: (1) outside and (2) within Flood Peak Periods (FPP).

In Fig 7 we show how further investigation into dependencies between combined tags ‘RW’ and flood-related tags outside (1a) and during (2a) flood peak periods respectively, this time after application of the magnitude condition, where impact is reflected by the number of crowd-generated content uploaded per day (e.g., more than 10, 50, 100 or 125 photos/videos). For comparison, we present the interaction between ‘RW’ and ‘NL’ tags outside (1b) and within (2b) flood peak periods. Here we can observe that the varying extent of the flood peak periods (FPP) has no effect on the tags’ relations outside these time intervals, and that the relationship between generic environmental and hydrological semantic elements are significantly stronger (*r* = 0.45, p[0.05], (1b)) than the relationships between ‘flood’ and ‘RW’ (*r* = 0.09, p[0.05], (1a)). Figs 2a and 2b demonstrate correlations within FPP and clearly illustrate that in these instances event magnitude really matters: For the pairs of hydrology- and risk-related tags, the correlation increases with the magnitude, and conversely, we can observe a simultaneous decrease for the tag pairs ‘NL’ and ‘RW’. In terms of temporal proximity to the peaks, we can conclude that combined tags can serve best as a predictor one day before the flood peak events, where statistically significant correlation drops to its minimum on the exact day of the maximum upload of UGC tagged with ‘flood’-related semantics.

**Fig 8:**
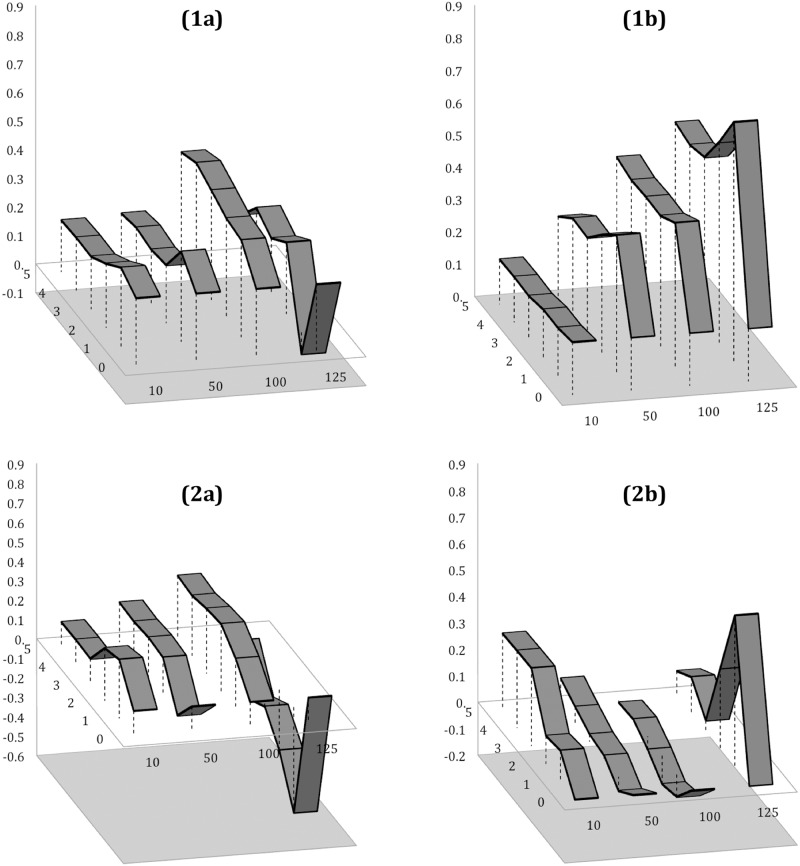
PPMCC values of the cross-dependencies between deconstructed ‘RW’ tag material: (a) ‘river’ vs. ‘flood’ (1a) and ‘NL’ (2a); and (b) ‘water’ vs. ‘flood’ (1b) and ‘NL’ (2b) within Flood Peak Periods (FPP) exclusively.

To investigate how each of the ‘RW’ components perform in conditions of varying magnitude, we focused on FPP only and compared relations between individual tags ‘river’, ‘water’ and risk-signalling tags (Fig 8(1a and 1b)) and combined nature-landscape thematics (Fig 8(2a and 2b)). We can observe straightaway that both scenarios are quite different for each hydrology-themed tag: Thus, the statistical performance of the ‘river’ tag differs very little when related to flood-signalling and landscape-neutral topics alike, while the strength of the relationship drops slightly in the case of ‘NL’ semantics (from *r* = 0.41 to *r* = 0.30, p[0.05]). The pattern of this relationship also demonstrates the identical ‘drop-effect’ when event magnitude increases to its highest band (more than 125 uploads per day), with the correlation coefficient being at its lowest on the day preceding the actual outbreak in both cases. We can also conclude that the main contribution to the correlative power between ‘RW’ and flood-related tags was predominantly due to the ‘water’ tag; thus Fig 8(1b and 2b) illustrates how correlation increases with event magnitude, peaking at its highest one day before the local maxima, and how the opposite to this trend effect is reflected in the case of ‘water’-‘NL’. We also observe unusual correlation behaviour for this pair of tags at the highest event magnitude level.

**Fig 9:**
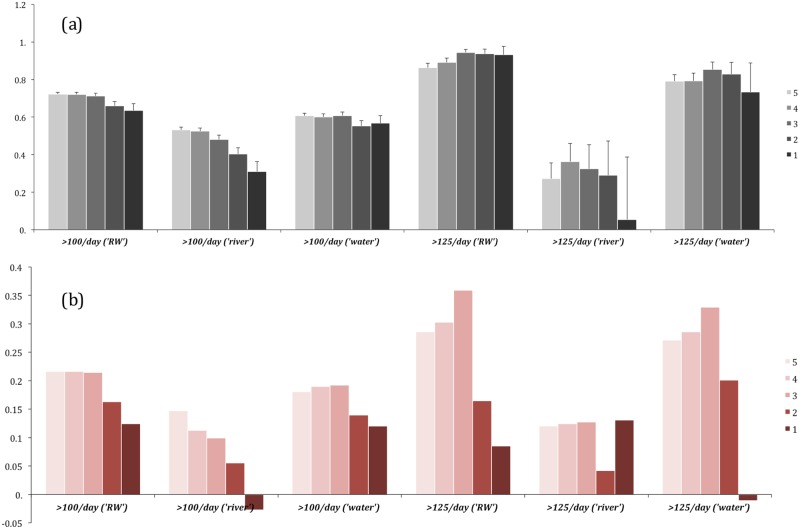
(a) PPMCC values of the cross-dependencies between the best performing predictor candidates (‘river’, ‘water’ and ‘RW’ tags) and hazard-signalling tags (‘flood’, ‘flooding’, ‘floodplain’) within 5-day time intervals before the event local maxima (more than 100 and 125 open source crowd-generated uploads to the Yahoo! Flickr platform); (b) PPMCC difference for the best predictor candidates when accounting for the pre-event interval only, as compared to the entire FPP.

Whilst we have successfully established connections between generic and risk-signalling environmental semantics, we still need to identify whether there is potential to forecast the flood event before its outbreak. Therefore, we reduce our time window to the five day pre-event interval and compare the dependencies across all best performing predictor candidates identified during the previous stages of the algorithm workflow and across event magnitudes for which they demonstrated the highest correlation capacity (more than 100 and 125 uploads of UGC per day). Fig 9a illustrates that narrowing the analytics temporal window throws the advance prediction timing of the best candidates ‘RW’ and ‘water’ tags back from one to three days. Fig 9b shows the difference in correlation power between the best selected candidates five days before the event outbreak and throughout the entire FPPs, which indicates a dramatic increase in the correlation power of the tag ‘water’ across both magnitudes (Fig 9b), thus producing a substantial peak in the aggregated ‘RW’ tag sequence three days before the event (on average, throughout the entire study period 2004–2014).

## Discussion

User generated content (UGC), i.e., any form of content such as blogs, wikis, discussion forums, posts, chats, tweets, images, video, audio files, etc., that is publically shared via social media platforms, is emerging as a valuable source of data for analyses requiring real-time insights into human behaviour, sentiment or mobility. At the same time and as a consequence of changing climate and increased urbanization, many countries are now exposed to an unprecedented number of natural hazards, which pose numerous threats to human health, life and economic development. Governments worldwide are concerned with climate threats and recognize the need for more enhanced natural environment monitoring systems, capable of predicting hazard occurrence, anticipation of its scale, extent and magnitude as well as mitigation of potential losses. As a consequence, new monitoring systems, largely consisting of sensors, gauges and stations, have been put in place in order to generate observation material to define and model various hazard scenarios. However, there is a growing concern that data flows produced by such earth-instrumentation systems cannot define and track natural hazards alone; rather they need to be combined with social and behavioural data reflecting how people are affected by the event at its various stages. There is therefore a need to look into how UGC, specifically its main characteristics of content, location and timestamps, can be used to enhance the capacity of natural environment monitoring systems to predict and mitigate the impact of such events.

Current applications of UGC in this context have focused on the use of timestamps and location metadata to derive useful knowledge about hazard development. Examples include the use of Yahoo! Flickr timestamps to nowcast ex-hurricane Sandy [4], use of Twitter during tsunami activity in Japan [3] and the very first application of Google Analytics to predict the flood impact in a number of UK cities during 2014–15 [20].

Our study therefore suggests a number of implications for the various disciplines involved, notably for computational hydrology, machine learning, Natural Language Processing and social sciences, and demonstrates how they can be combined together in order to solve highly interdisciplinary problems.

Firstly, social media analytical frameworks could greatly benefit from closer integration with hydrological and meteorological models; by combining both sources of data we could produce more flexible and more locally (both socially and environmentally) adapted warning notice periods, and, as a consequence, quantify the inevitable impact of hazards and define local resilience thresholds [21].

This type of context-based analytics could also contribute to certain areas of Natural Language Processing, notably connotation detection. Automated word-sense disambiguation (WSD) is currently defined as an open problem in computational linguistics and ontology [22] as connotation, as opposed to sentiment, is a more subtle form of meaning, combining cultural, emotional and contextual associations [23, 24]. As a result, connotation can be evoked by words that do not express sentiment and that would be considered neutral in sentiment analysis or opinion mining tasks. This issue could be approached from the evolving context perspective and linked to other environmental data sources, which could help to detect connotation change triggers. Conversely, NLP techniques could be further exploited in order to increase the lexical pool of potential candidates, which could be defined computationally on a basis of their semantic properties.

The best predictor estimation learning algorithm was applied in this study to a static dataset, however, there is a scope for subsequent adaptations into real-time machine learning tools, capable of reading, combining and interpreting real-time data from several sources, including sensor readings. A new generation of adaptive forecasting systems opens challenges not only for soft computing, but equally for combined software/hardware configurations [25], which will need to support integrated processing of heterogeneous real-time data.

## Conclusion

From this study we conclude that alternative environmental semantics derived from ‘social sensors’ can be used as a hazard predictor and, in the case of flooding, combined use of hydrological terms with more generic (e.g., ‘water’) and more origin-specific (e.g., ‘river’, ‘lake’, ‘watercourse’) meanings can provide the best results during high impact, high resonance events.

The findings of this study have a high significance and represent a substantial leap in the field of the application of social media analytics in the natural sciences. Further investigations in this direction are recommended in order to assist design and implementation of socially adapted hazard warning systems, however, other similar applications could also be identified and tested.
